# Porcine Wharton’s jelly cells distribute throughout the body after intraperitoneal injection

**DOI:** 10.1186/s13287-018-0775-7

**Published:** 2018-02-14

**Authors:** Kreeson Packthongsuk, Theresa Rathbun, Deryl Troyer, Duane L. Davis

**Affiliations:** 1 National Institute of Animal Health (NIAH) 50/2 Kasetklang, Pahonyothin Rd., Jatujak, Ladyao, Bangkok, 10900 Thailand; 2 Department of Animal Sciences and Industry, Kansas State University, Manhattan, 66506 KS USA; 3 Department of Anatomy and Physiology, Kansas State University, Manhattan, 66506 KS USA

**Keywords:** Cell transplantation, Mesenchymal stem cells, Pig, Umbilical cord, Wharton’s jelly cells

## Abstract

**Background:**

Wharton's jelly cells (WJCs) have multiple differentiation potentials and are easily harvested in large numbers. WJCs are well tolerated in allogeneic environments and there is a growing list of their therapeutic effects. Most therapies require administering large numbers of cells and this is generally accomplished by intravenous injection. Here, we studied the locations of porcine WJCs in immune-competent, allogeneic hosts after intraperitoneal (IP) injection.

**Methods:**

Male porcine WJCs were administered to female neonatal piglets by IP injection. The location of transplanted cells was examined at 6 h, 24 h, and 7 days after administration using confocal microscopy and polymerase chain reaction (PCR). Transplanted cells were also retrieved from the intestines of recipients and were cultured. Previously transplanted cells were identified by fluorescence in-situ hybridization (FISH) using a Y-chromosome probe.

**Results:**

Allogeneic cells were identified in the small and large intestine, stomach, liver, spleen, diaphragm, omentum, kidney, pancreas, mesenteric lymph nodes, heart, lungs, uterus, bladder, and skeletal muscle. Male cells (SRY positive) were found in cultures of cells harvested from the intestinal mucosa 1 week after administration of male porcine WJCs.

**Conclusions:**

Our results show that porcine WJCs distribute widely to the organs in immunocompetent allogeneic hosts after IP administration. They may distribute through the lymphatics initially, and a prominent site of incorporation is the mucosa of the gastrointestinal tract. In that location they could function in the niche of endogenous stem cells and provide secretory products to cells in the tissue damaged by intestinal disease.

**Electronic supplementary material:**

The online version of this article (10.1186/s13287-018-0775-7) contains supplementary material, which is available to authorized users.

## Background

Mesenchymal stromal (stem) cells (MSCs) have been isolated from many tissues and have broad therapeutic potential. Their effects include immune regulation and chemokine and cytokine production [1–3]. MSCs are currently the subject of clinical trials for a range of conditions that would benefit from their cell and tissue regenerative, trophic, immune modulating, and chemo-attraction properties. Wharton’s jelly is a prenatal source of MSCs (WJCs) that are easily collected at birth. WJCs are available in large numbers and are tolerated in allogeneic recipients even when purposely mismatched for major histocompatibility complex [4]. WJCs lack tumorgenicity, are anti-inflammatory, and have anti-cancer effects [5]. These characteristics make them attractive candidates for transplantation therapies.

Transplant recipients are generally given millions of WJCs and other MSCs by intravenous (IV) injections. There are potential drawbacks for IV administration, including retention of cells in the lungs [6, 7]. Intraperitoneal (IP) administration is an alternative route of injection, but a recent report [8] found that human MSCs given IP to mice resulted in their aggregation with macrophages and B220^+^ lymphocytes. The aggregates attached to the wall of the peritoneal cavity and the authors concluded that the aggregated MSCs probably had limited access to the systemic circulation. Bazhanov et al. [8] studied xenogeneic transplants of human bone marrow-derived MSCs injected IP in mice, and those aspects of their model could have affected results. Here, we show that allogeneic porcine WJCs injected IP distribute to a wide variety of tissues both within and outside the peritoneal cavity and can be recovered from the intestinal mucosa and re-cultured 1 week after transplant.

## Methods

### Animals and cell collection

Animal procedures were approved by the Kansas State University Institutional Animal Care and Use Committee (protocol no. 3055). Umbilical cords were collected from pigs (genetic lines 1050 × 327, PIC North America, Hendersonville, TN, USA). At farrowing, pigs were held as they emerged from the vulva and the umbilical cord was collected taking care they did not contact the sow’s skin or other surfaces. Umbilical cords were transported to the laboratory in phosphate-buffered saline (PBS) supplemented with penicillin (100 μg/mL), streptomycin (100 μg/mL), and amphotericin (0.25 μg/mL) (Gibco®, Grand Island, NY, USA). The vein and arteries were removed with forceps and discarded with the adherent perivascular jelly. The remaining gelatinous tissue was removed from the amnion by scraping and minced into pieces (1–3 mm^3^). The pieces of jelly were placed in culture dishes and allowed to air-dry (10 min) to increase adhesion to the plastic. Then growth medium was added. Pig umbilical cords have small vascular structures throughout the jelly [9]. These structures do not persist and by the second-passage WJC culture were homogeneously mesenchymal.

### Cell culture

The growth medium was high-glucose Dulbecco’s minimum essential medium (DMEM) with HEPES (GlutaMAX™-DMEM-HEPES) supplemented with fetal bovine serum (FBS; 20%), gentamicin (25 μg/mL) (all from Invitrogen, Carlsbad, CA, USA), Normocin™ (100 μg/mL; Invivogen, San Diego, CA, USA), and 2-mercaptoethanol (55 μmol/mL; Sigma-Aldrich, St. Louis, MO, USA). Cultures were incubated (38.5 °C in 5% CO_2_:95% air and saturated humidity) and explants removed after cells migrated from the tissue (10–14 days). When 80% confluent, cells were harvested (0.05% trypsin/EDTA; Gibco®). Cells were either re-plated in growth medium or cryopreserved in recovery cell culture freezing medium (FM; Gibco®). Cell concentrations and viability were routinely determined using a microcapillary flow cytometer (Guava Viacount® reagent and Guava EasyCyte Plus; Millipore, Billerica, MA, USA). Cells were used at passage 5 or less for all experiments.

### Characterization of porcine WJCs

Adipogenesis, chondrogenesis, and osteogenesis were tested with StemPro® kits (Gibco) which were used according to the manufacturer’s instructions (Additional file 1).

The immunophenotype of porcine WJCs was analyzed using mouse monoclonal antibodies to porcine CD31 (LCI-4):IgG1-RPE, CD45 (K252.1E4):IgG1-FITC, and SLA class II DR (2E9/13):IgG2b-FITC (AbD Serotec, Bio-Rad, Hercules, CA, USA), porcine CD105 (MEM-229):IgG2a-FITC and CD90 (5E10):IgG1-FITC (Abcam, Cambridge, MA, USA), and porcine CD44 (MEM-263):IgG1-FITC (Thermo Scientific, Middletown, VA, USA). All isotype control antibodies were derived from mice (IgG1-FITC, IgG1-RPE, IgG2a-FITC, and IgG2b-FITC) and were purchased from Invitrogen (Carlsbad, CA, USA). Antibodies for porcine CD73 were not available and monoclonal antibodies against human CD73 do not cross-react with porcine CD73 [10, 11]. Cell suspensions (1 × 10^7^ cells/ml in 100 μL PBS) were incubated with antibodies or isotype controls (final concentration 3 μg/mL) for 45 min at 4 °C in the dark. Fluorescence was analyzed with a microcapillary cytometer (Guava EasyCyte Plus and Cytosoft™ software, Millipore).

### Administration of cells to piglets

Cells were labeled with either PKH26GL (Sigma Aldrich) or CellVue (LI COR Biosciences, Lincoln, NE, USA) according to the manufacturers’ instructions (Additional file 2).

In experiment 1 we determined the distribution of cells soon after intraperitoneal (IP) injection. Four piglets were given dye-labeled cells within 1 h of birth and two were sacrificed at 6 h and two at 24 h of age. In experiment 2 we evaluated the location of labeled cells 1 week after IP administration at birth, 1 day, 1 week, 2 weeks, or 3 weeks of age. Two pigs were treated IP at each age. In addition, three piglets were given only culture medium without cells at birth. These controls were sacrificed 7 days after administering the culture medium.

For IP administration, piglets were restrained by holding the rear legs with the head down. The labeled cells (1 × 10^6^) were injected in 3 mL of medium into the peritoneal cavity using an 18 gauge 1–1/2” needle placed on the right of the teat line between the first and the second pairs of nipples. The piglets were returned to the sow and observed for 30 min. Piglets were sacrificed after inhalation of isoflurane (1–5% in O_2_) to deep anesthesia followed by intracardiac injection of a saturated solution KCl to induce cardiac arrest.

### Tissue collection

Blood samples (10 mL) were collected by cardiac puncture prior to KCl injection and transferred to tubes containing sodium heparin. Following sacrifice, tissues were collected from the heart, lung, pancreas, liver, kidney, omentum, stomach, intestine, uterine horn, ovary, muscle, and bone marrow. The whole intestine was opened longitudinally, and the intestinal lumen washed with tap water and kept in ice cold 1× HBSS containing Ca^2+^ and Mg^2+^. The intestines were 350–430 cm long and samples were selected throughout the length of the jejunum, ileum, duodenum, and colon for polymerase chain reaction (PCR) and confocal analysis. Tissues were preserved in DMSO/EDTA/saturated NaCl buffer (DESS) buffer and 4% paraformaldehyde solution (16%; Thermo Scientific) for cryosection and DNA isolation.

Blood was diluted 1:1 with PBS, and the cell pellet separated by centrifugation (400 × g, 8 min) and re-suspended in 10 mL of red blood cell lysis buffer for 10 min followed by washing (2×) with PBS.

Femurs were cleaned of connective tissues and the ends opened to expose the marrow. Femurs were flushed from both ends by injecting DMEM + EDTA (5 mM) and cells were recovered by centrifugation (400 × g, 8 min), suspended in red-blood cell lysis buffer (Sigma) for 5 min, and washed twice with PBS. Cells and tissues were evaluated for the Y-chromosome gene SRY by PCR. Presence of transplanted PKH26-labeled WJCs was detected by microcapillary cytometry.

### PCR

Samples (25 mg) of tissues collected from the recipients and stored in DESS were homogenized and total DNA was isolated using DNeasy Kits (Qiagen, Germantown, MD, USA). Genomic DNA (2 μg) was used as the template for 50-μL PCR reactions using the HotStar Taq DNA Polymerase Kit (Qiagen). PCR reactions contained 1× PCR buffer, 2.0 mM MgCl_2_, 0.8 mM dNTP (Advantage Ultrapure Nucleotide, Clonetech), 1× Q solution, 0.05 U/μl of HotStart Taq DNA polymerase, and 0.28 μM specific porcine SRY forward and reverse primers (forward: 5’-GGCTTTCTGTTCCTGAGCAC-3’; reverse: 5’-CTGGGATGCAAGTGGAAAAT-3’; product size: 247 bp) and porcine-specific beta-actin genes forward and reverse primer (forward: 5’-GTCATCACCATCGGCAATGA-3’; reverse: 5’-CGTGAATGCCGCAGGATT-3’; product size: 183 bp). The PCR conditions were 1 cycle (15 min at 95 °C) followed by 35 cycles (30 s at 95 °C) followed by 30 s (59 °C), 30 s (72 °C), and finishing with 7-min incubation at 72 °C. Reaction products were stained with ethidium bromide (250 ng/μl) and visualized by agarose gel electrophoresis (2.5%).

### Cells from the intestine for culture and fluorescent in-situ hybridization (FISH)

Pieces of the intestine (15–20 cm) were inverted, cleaned, and kept in ice-cold DMEM without serum until cell isolation when they were incubated in PBS with EDTA (1 mmol/L) and dithiothreitol (1 mmol/L) without Ca^2+^ and Mg^2+^ (7 min at 37 °C). After washing twice (5 min in PBS without Ca^2+^ and Mg^2+^) they were digested in collagenase type 3 (220 U/mL), neutral protease (0.5 U/mL), and deoxyribonuclease type 1 (50 U/mL) (all enzymes were from Worthington) in growth medium with shaking (low speed, 37 °C for 45 min). The enzymatic reactions were inactivated (4 °C for 5 min) and the cell suspension filtered (Steriflip® 60-μm pore size; EMD Millipore), and centrifuged (500 × g for 10 min, 4 °C). The cell pellet was washed twice with PBS and cells were plated on chamber slides (Lab-Tek® II Slide) and a tissue culture flask (75 cm^2^) with growth medium.

After culture for 7–10 days (medium replaced every 3 days) the slides were washed (3× PBS), fixed with cold methanol:glacial acetic acid (3:1), washed in PBS (3×, 5 min), and incubated in 10 mM sodium citrate (pH 6.0) at 96 °C for 15 min. Slides were air-dried (5 min room temperature (RT)) and washed twice (5 min) in 2× SSC (0.15 mol/L sodium chloride, 0.015 mol/L sodium citrate, RT). Subsequently, the slides were treated with 200 μL RNase A (100 μg/mL; Sigma) in 2× SSC for 1 h at 37 °C and washed twice (5 min in 2× SSC solution at RT). Then, the slides were rinsed with 10 mM HCl (2 min) and treated with 200 μl 40 U/mL pepsin (Sigma) in 10 mM HCl for 10 min at 37 °C. The slides were rinsed with distilled water and washed twice for 5 min (2× SSC solution, RT). The slides were dehydrated by passing through a series of ethanol concentrations (70%, 85%, 100%) and air-dried. Chromosome denaturation was carried out in 70% (v/v) formamide/2× SSC solution at 75 °C for 5 min.

A Hybriwell™ (Sigma) slide sealing system was installed over the slide and filled with a hybridization mixture (24 μL purified digoxigenin (DIG)-labeled DNA probe solution from two SRY PCR reactions and 126 μl hybridization buffer) and sealed with adhesive plastic. Next, the slide was incubated (75 °C, 5 min) to denature DIG-labeled DNA probes and then incubated in a dark moist chamber (37 °C, 24 h) for probe hybridization. The Hybriwell was removed and excess probe removed with two 5-min incubations in SSC. The slide was incubated in 50% formamide/2× SSC solution for 2 min (42 °C) and washed (2× SSC buffer and in 1× SCC buffer, 15 min each at 42 °C) followed by two washes in 0.5× SSC buffer (each 10 min).

After washing three times in PBS (5 min) the slide was blocked for nonspecific binding (1% normal goat serum and 0.1% Triton X-100 in PBS for 30 min). The blocking solution was replaced with 200 μL conjugation buffer (1:15 conjugate stock solution and blocking solution) for 12 h at 4 °C, washed (PBS 3×, 5 min), and rinsed twice with distilled water. Cell nuclei were counterstained with Syto®16 (Invitrogen) for 8 min and mounted with glycerol for confocal microscopy. Detection of DIG-labeled DNA hybridized probes was performed by direct fluorescence detection. Fab fragments from an anti-DIG antibody conjugated with 5-carboxy-tetramethyl-rhodamine-N-hydroxy-succinimide ester (TAMRA; Roche) with maximum excitation/emission as 555/580 bound directly and specifically to DIG-labeled DNA probes.

### Synthesis of DIG-labeled DNA probes

Based on PCR reaction, 377 bp of dsDNA probe specific to porcine Y chromosome SRY was synthesized and labeled with DIG using a PCR DIG Probe Synthesis Kit (Roche) according to the manufacturer’s instructions. Briefly, 50 ng of male genomic DNA isolated from male WJCs was added to a 50-μL PCR reaction containing 1× PCR buffer with 1.5 mmol/L MgCl_2_, 1× PCR DIG Probe Synthesis Mix containing dNTPs and DIG-11-dUTP, 0.75 μL of enzyme mix, and 0.25 μmol/L of specific porcine SRY forward and reverse primers (forward: 5’-AATCCACCATACCTCATGGACC-3’; reverse: 5’-TTTCTCCTGTATCCTCCTGC-3’; product size: 377 bp). PCR conditions were 1 cycle of 2 min at 95 °C, followed by 35 cycles of 15 s at 95 °C, 60 s at 60 °C, and 15 s at 72 °C, and finishing with 7-min incubation at 72 °C. DIG-labeled DNA probes were isolated and purified from PCR reactions using QIAquick PCR Purification Kit (Qiagen) according to the manufacturer’s instructions. PCR reactions were treated with high chaotropic salt buffer and ethanol and purified in spin columns. Size and purity of eluted DIG-labeled dsDNA probes were determined by staining with ethidium bromide and visualized by agarose gel electrophoresis.

### Confocal microscopy

Fixed tissues were washed, incubated in 30% sucrose (w/v) in PBS (24 h at 4 °C), treated with cryoprotectant and submerged in cryomolds with embedding medium (Tissue-Tek®), frozen in liquid nitrogen, and stored (–70 °C). Sections (12 μm) were examined using an inverted Zeiss LSM 700 confocal microscope (LSM 700, Carl Zeiss). Plan-Neofluar objectives (20×/0.50 and 40×/1.30 Oil M27 EC) with a pinhole (33 μm).

### Statistical analysis

The proportion of tissues samples positive for SRY was evaluated using Procedure GLM of SAS (SAS Institute, Inc., Cary, NC) and, when the F-test was significant (*P* < 0.05), means were compared using PDIFF.

## Results

### Mesodermal differentiation and immunophenotype of WJCs

After 14–21 days in the differentiation media WJCs demonstrated adipogenic, osteogenic, and chondrogenic differentiation as assessed by appropriate staining (Additional file 3: Figure S1 and Additional file 4: Figure S2) as expected for MSCs [12]. Flow cytometry results showed that WJCs displayed the immunophenotype of MSCs. They were positive (>90%) for the stromal markers CD44, CD90, and CD105, but < 2% expressed the hematopoietic markers CD45, CD31, and HLA-DR (Additional file 5: Figure S3 and Additional file 6: Table S1).

### Transplantation of labeled WJCs

For initial IP transplants, the cells were administered in medium with serum (20% FBS) and two of 16 recipients transplanted IP showed symptoms of anaphylaxis with labored breathing, vomiting, and pale skin within 10 min. Both appeared to recover within 1 h but one piglet died within 24 h. The other piglet exhibited no further symptoms. Subsequent IP transplantations (three recipients) were in medium without serum and these symptoms were not observed.

#### Experiment 1

PCR analysis for SRY indicated that allogeneic male WJCs were present in some samples from all the tissues collected after IP injection and sacrifice at 6 (*n* = 2) and 24 h (*n* = 2) after transplantation. At the earliest sacrifice (6 h), SRY was detected in all samples from the omentum and diaphragm (Table 1). Over all organs there was a trend for more samples to be positive for SRY by 24 h after transplantation and both pigs had positive samples in all organs. Overall, 53% of the tissue samples were positive for SRY and there were positive samples for all tissues for all piglets. Cells isolated from blood were not checked for the presence of SRY, and PKH fluorescing cells were not detected in blood by flow cytometry.

**Table 1:** Identification of male WJCs in pigs receiving intraperitoneal transplants at birth and sacrificed 6 or 24 h later

	Sacrificed at 6 h			Sacrificed at 24 h		
	Pig A	Pig B	Pigs A + B	Pig C	Pig D	Pigs C + D
Tissue samples examined for Sry/tissue/pig	3	3	6	3	3	6
Diaphragm	3^a^	3	6	3	3	6
Omentum	3	3	6	3	3	6
Pancreas	2	1	3	2	2	4
Spleen	1	2	3	2	2	4
Ileum	1	1	2	3	2	5
Jejunum	1	2	3	2	2	4
Duodenum	1	1	2	2	2	4
Stomach	2	1	3	2	1	3
Cecum	1	1	2	2	1	3
Colon	2	0	2	2	2	4
Heart	1	1	2	1	2	3
Liver	1	1	2	2	3	2
Kidney	0	1	1	1	1	2
MLNs	0	1	1	1	1	2
Lung	1	1	2	1	1	2

*MLNs* mesenteric lymph nodes

^a^Positive samples for each pig

#### Experiment 2

**Fig. 1:**
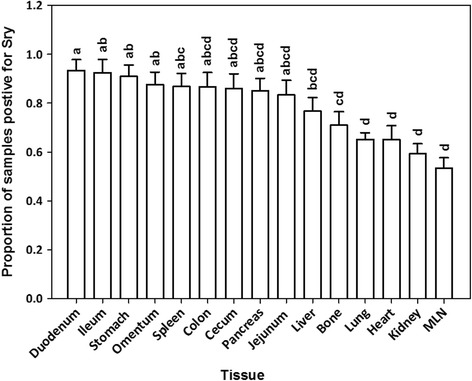
SRY-positive samples 1 week after intraperitoneal transplantation. WJCs were administered IP to 1-, 7-, 14-, and 21-day-old pigs (2/day). The piglets were sacrificed 1 week later and four samples were collected from each organ of the 1-day-old recipients and three samples per organ from the other age groups. Each bar represents the mean proportion of samples from each eight female piglets for each organ for which PCR detected the SRY gene indicating the presence of transplanted male WJCs. Bars without the same superscript differ (*P* < 0.05). There was no effect (*P* > 0.9) of age at transplant or age × organ interaction. MLN mesenteric lymph node

**Fig. 2:**
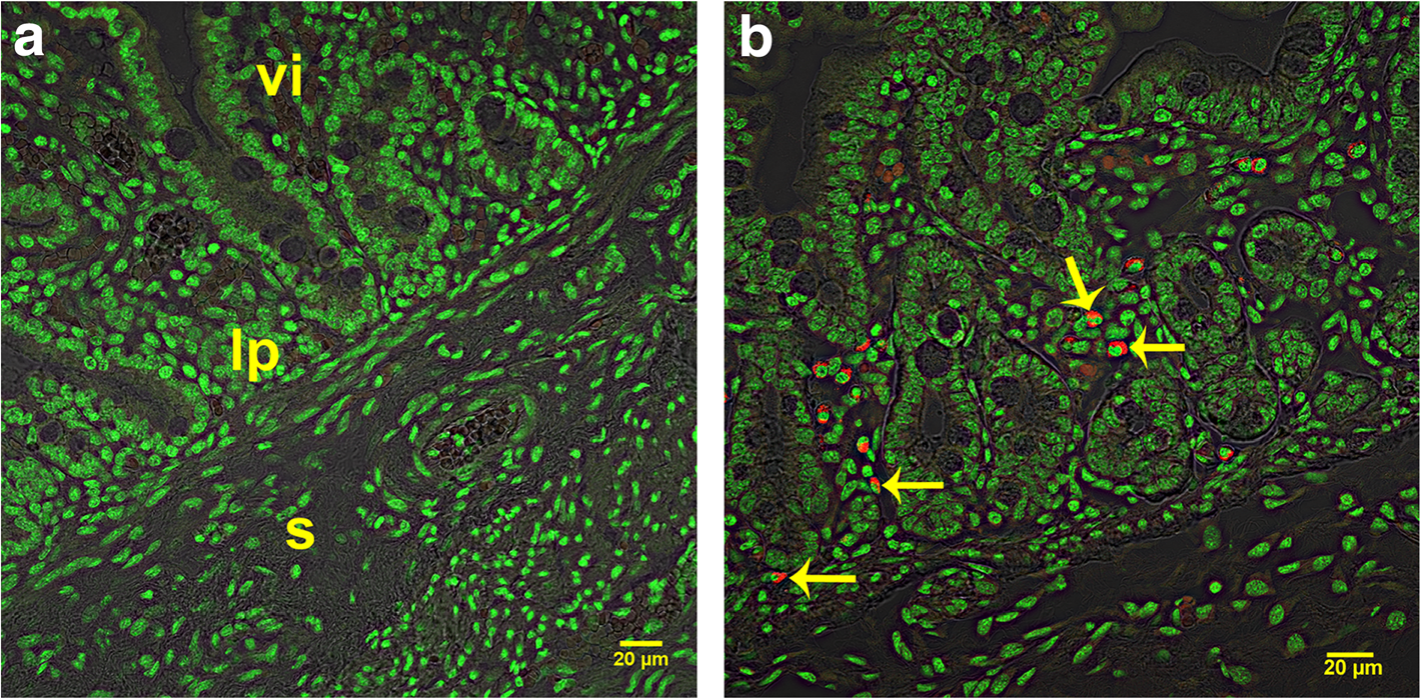
Confocal images illustrating intestine cryosections 1 week after transplantation. **a** Duodenal cryosection from a control (nontransplanted) piglet. **b** Cryosection from the duodenum of a piglet sacrificed 1 week after transplantation of PKH26-labeled WJCs by IP injection. Numerous PKH26-labeled (red) cells are visible in the stromal layer underlying the epithelium (arrows). s submucosal layer, lp lamina propria, vi villus

**Fig. 3:**
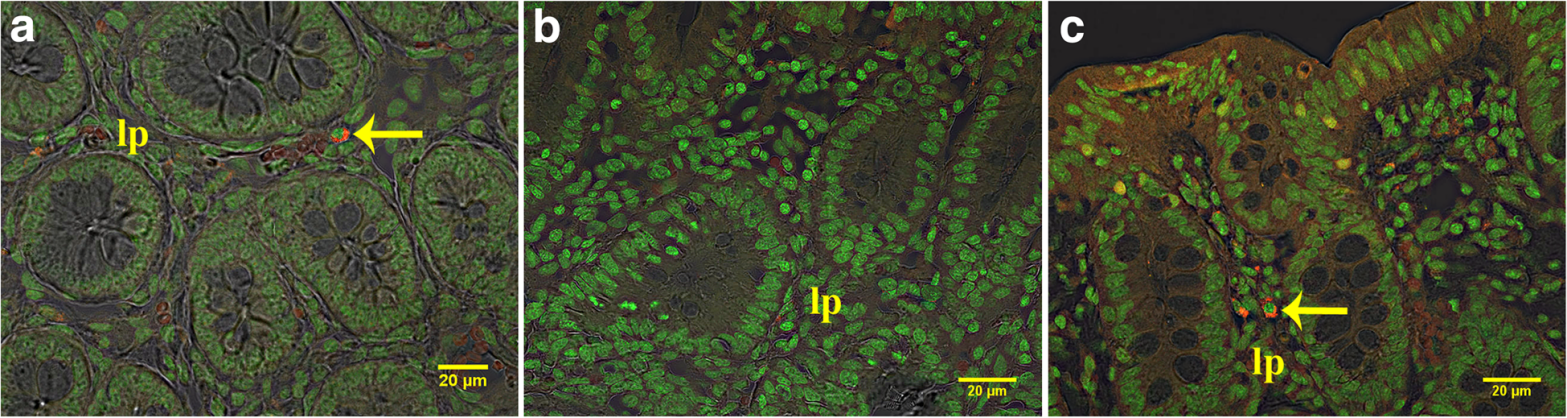
Confocal images of cecal and colon cryosections from 1-week-old piglets given cells IP at birth. **a** Cryosection from the cecum of a piglet receiving PKH26-labeled WJCs. **b** The colon from a control pig and **c** colon of 1-week-old piglet after IP injection of PKH26-labeled WJCs at birth. Arrows indicate PKH26-labeled (red) cells. lp lamina propria

**Fig. 4:**
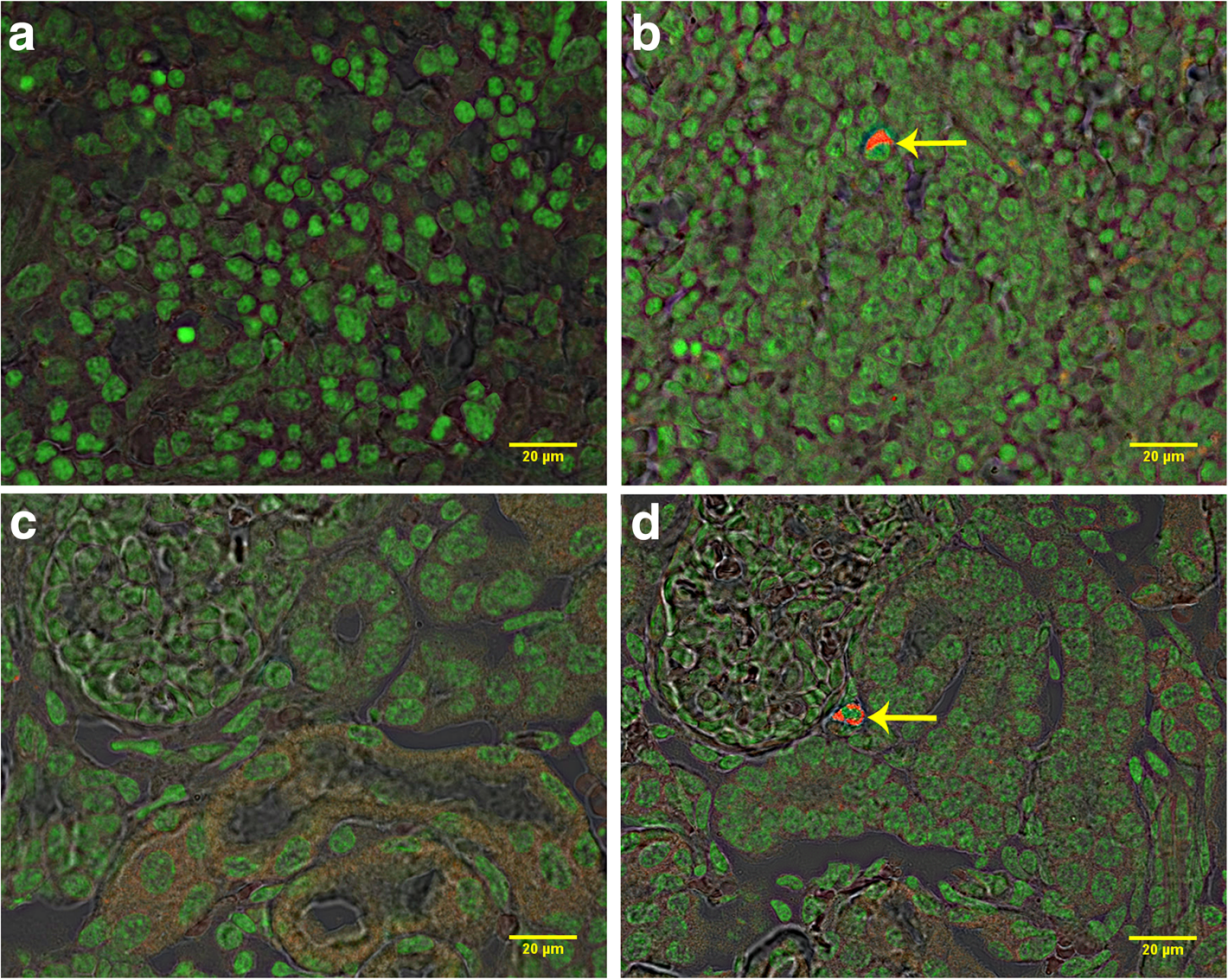
Confocal images of cryosections of spleen and kidney from control and transplanted piglets injected at 1 day of age and sacrificed 7 days later. **a** Spleen of control piglet and **b** transplanted piglet. **c** Kidney of control newborn piglet. **d** Kidney from a transplanted piglet. Arrows indicate PKH26-labeled (red) cells

**Fig. 5:**
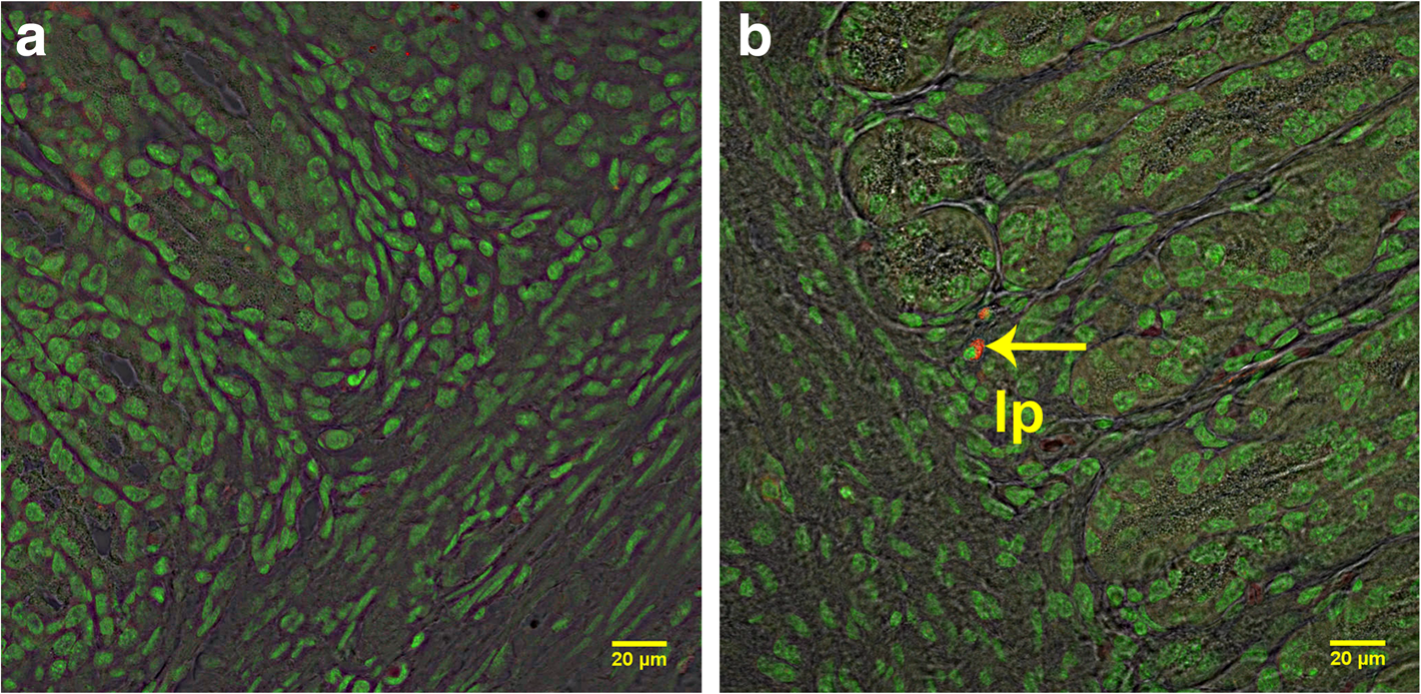
Confocal images of stomach cryosections of control and transplanted piglets. Stomachs from 1-week-old piglets. **a** Control and **b** piglet transplanted with PKH26-labeled WJCs at birth. Arrows indicate PKH26-labeled (red) cells. lp lamina propria

With the exception of blood, all tissues were positive for the SRY gene for 1-day-, 1-week-, 2-week-, and 3-week-old recipients (Fig. 1). SRY was detected in every organ in every recipient piglet. Therefore, the cells consistently distributed broadly after IP transplant. The percentage of positive samples in each organ provides a semi-quantitative measure of the location of the transplanted WJC and was affected by organ (*P* < 0.0001) but not by age at transplant (*P* > 0.9) or the interaction of age × organ (*P* > 0.9). The percentage of positive samples ranged from 53 to 96%. At least 85% of samples from the small intestine, stomach, spleen, and omentum were positive and this was greater (*P* < 0.05) than the 54 to 65% of samples from the lung, heart, kidney, and mesenteric lymph nodes. The other organs tested were intermediate to these. Samples were also evaluated for the uterine horns and bladder for four piglets and the semimembranosus muscle for two piglets (Additional file 7: Table S2). PCR revealed that 67% of these samples were SRY-positive. Cryosections of organs collected from newborn piglet recipients of PKH26-labeled WJCs demonstrated PKH26-labeled cells. A major site of incorporation is the mucosal layer of the intestine (Fig. 2) where the WJCs were predominantly at the base of villi and the area around the crypts. WJCs were observed in the large intestine (Fig. 3b) at similar sites. Labeled cells were also detected in cryosections of spleen, kidney, and stomach (Fig. 4). There were no cells containing red fluorescence in cryosections obtained from controls (Figs. 2a, 3b, 4a, and 5a).

**Fig. 6:**
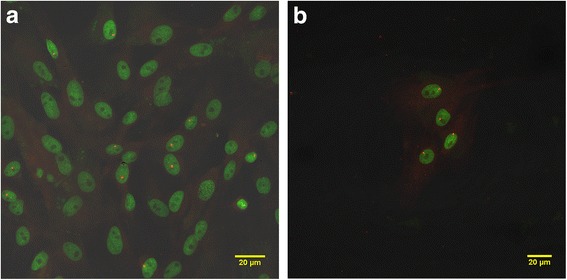
Confocal images of cells isolated from the submucosa of intestines of female recipients 1 week after IP transplant. Male transplanted WJCs are identified by FISH. Nuclei are stained green with Syto®16 and yellow spots indicate the labeled SRY gene on the Y chromosome (**a**). A colony of allogeneic male WJCs (**b**) isolated from the intestine of a transplant recipient

**Fig. 7:**
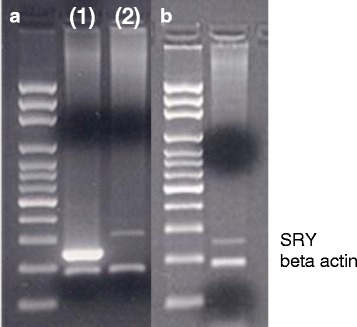
PCR detection of the SRY gene in an extract of cells derived from the intestine of a female recipient. Agarose gel electrophoresis showing a PCR product of the SRY gene (247 bp). Male (1) and female (2) control (**a**) and engrafted male WJCs (**b**) recovered from the ileum of 2-week-old female porcine recipient 1 week after IP transplantation of male porcine WJCs. A lower PCR product (183 bp) is the porcine-specific beta actin gene used as an internal control. The left lanes are a 100-bp DNA ladder

Intestinal cells from IP-treated WJCs of all ages were isolated by enzymatic digestion and cultured in vitro. After plating, attachment of isolated intestinal cells was observed by 2 days and colonies formed within 10 days. After 2 weeks of growth, FISH analysis revealed the presence of SRY-positive cells identifying male WJCs (Table 2 and Fig. 6). Colonies of donor male WJCs also were detected by FISH when intestinal cells were plated at low cell density (Fig. 6b). PCR confirmed the presence of the SRY gene in these cultured intestinal cells (Fig. 7).

**Table 2:** Results of culture of intestinal cells isolated from small intestines of female porcine recipients 1 week after transplantation

Detection methods	Age of female pigs at transplantation					
	Newborn (*n* = 2)		1 day (*n* = 2)	1 week (*n* = 2)	2 weeks (*n* = 2)	3 weeks (*n* = 2)
	Oral	IP	IP	IP	IP	IP
Samples with Sry DNA						
PCR	1,1^a^	1,1	1,1	1,1	1,1	1,1
Samples with detected Sry probes						
Fluorescent in-situ hybridization (FISH)	1,1	1,1	ND	1,Cont	1,1	1,1

*Cont* microbial contamination, *IP* intraperitoneal, *ND* not done, *PCR* polymerase chain reaction

^a^No. of positive samples for each recipient

## Discussion

The MSCs in Wharton’s jelly in the umbilical cord can be harvested without ethical concerns and they have the potential for a wide range of therapies [13]. MSCs have been the subject of numerous preclinical and clinical trials [14, 15] and their therapeutic potential extends to a variety of tissue targets that correspond to the sites we observed to harbor allogeneic WJCs after IP injection.

By transplanting male WJCs into female piglets we were able to monitor the distribution of transplanted allogeneic cells using PCR to detect the SRY gene. Pigs are a good species for cell tracking in this way because prenatal chimerism does not occur in pigs [16]. Therefore, the SRY-positive cells indicate the locations of transplanted cells or their progeny. Our results show that allogeneic pig WJCs injected IP consistently reach tissues throughout the body. This result indicates that IP injection should be considered in WJC transplantations. As recently pointed out by Parys et al. [17] the IP route limits the potential side effects that have been reported after intravenous injections of MSCs. These effects include retention of cells in the lungs [6, 7] and an instant blood-mediated inflammatory reaction [18]. It has also been reported that exposure to the lung environment alters the expression of genes regulating immunological pathways [19].

Our results contrast with the report of Bazhanov et al. [8] who found that human bone marrow-derived MSCs appeared to aggregate with macrophages and B220^+^ lymphocytes in the peritoneal cavity of mice and did not reach other sites. Perhaps consistent with Bazhanov et al., Mauri et al. [20] transplanted bone marrow-derived mouse MSCs into mice and did not detect the IP injected MSCs in the lungs, spleen, or liver 24 h after injection. They did, however, demonstrate improvement in lung functions in the transplanted mice after acute injury, and thus effects could have been due to paracrine mechanisms. As pointed out by Bazhanov et al., others have reported distribution of MSCs into various tissues after IP administration of human adipose-derived MSCs into an immunotolerant mouse [21] or IP administration of bone marrow-derived Sprague-Dawley rat MSCs into siblings of the donor [22].

Methodological differences between our experiments and those of Bazhanov et al. [8] are their use of xenogeneic MSCs (human in mouse), their use of bone marrow MSCs as opposed to WJCs in our studies, and our use of the allogeneic pig model as opposed to the human-mouse xenogeneic model by Bazhanov et al.

There are differences between species regarding MSC function. For example, mouse, rat, rabbit, and hamster MSCs use nitric oxide as an immunosuppressive signal, but human, monkey, and pig MSCs utilize indoleamine-2,3-dioxygenase to suppress immune responses [23]. Regarding the type of MSC, WJCs conform to the general characteristics of MSCs and are plastic-adherent, display mesodermal differentiation and stromal cell markers, and lack hematopoietic markers. We confirmed these characteristics for pig WJCs in our preliminary studies. However, WJCs appear to be intermediate between embryonic and adult stem cells and have greater multipotent plasticity and proliferate at higher rates than MSCs from adult tissues [24, 25]. How the choice of MSC type or species may affect the results of IP transplant experiments will require further investigation.

Transplantation of MSCs has mostly been evaluated in xenogeneic models of human diseases. The hosts are generally immune-compromised and often these models include transplanted tumors or have damaged organs. These models show that WJCs and MSCs home to pathologies at least in part by responding to inflammatory chemokines [26, 27]. In our studies, we demonstrated the ability of allogeneic porcine WJCs to migrate from their initial location at administration in the peritoneal cavity into healthy tissues throughout the body of immunocompetent piglets. These sites would also likely be accessed by greater numbers of transplanted WJCs if inflammation existed in these sites and perhaps in greater numbers due to the homing response to inflammation.

At 6 h after IP transplantation, male WJCs were detected in the diaphragm and omentum in all samples examined. After IP administration these tissues appear to be a part of the early transit of WJCs and they might reach the diaphragmatic lymph system through peritoneal stomata [28] and the mesothelial stoma overlying milky spots in the omentum [29, 30]. Once in the lymph, cells could pass through the thoracic duct and into the blood [31]. After accessing the intestinal lymphatics and the abdominal lymph flow the IP-administered porcine WJCs could have achieved general distribution through lymphatic and then vascular routes.

A novel aspect of the current work is the recovery of transplanted WJCs from the intestinal mucosa of recipients. Isolation and re-culture of transplanted cells is feasible because mesenchymal cells are plastic-adherent and can be plated at low density to separate colony-founding cells. We isolated and cultured the transplanted WJCs from the intestinal mucosa 7 days after transplant. The recovered cells were identified as male by the SRY gene after analysis by PCR and FISH. Because they were plastic-adherent and formed colonies they have characteristics similar to the cells before transplant. These results suggest the cells are viable and mitotically competent after transplant.

The colonies formed in vitro by recovered porcine WJCs provide a visualization of the possible impact of the distribution of the transplanted cells. Although we have no direct evidence of this behavior in vivo, if it does occur then once incorporated into host tissues, WJCs might divide and produce similar colonies where their influence could have an impact particularly in the stromal layers of tissues.

The identification of transplanted porcine WJCs in the gut may be particularly important. Endogenous gut MSCs appear to have critical roles in homeostasis and immunity [32] and MSCs may not persist but exert many of their effects as immune and inflammatory regulators [32]. The maintenance of mesenchymal phenotype and residence in the gut mucosa of allogeneic porcine WJCs is important since clinical trials indicate beneficial effects of systemically administered MSCs in patients with refractory Crohn’s disease [33].

## Conclusions

Allogeneic porcine WJCs transplanted to the peritoneal cavity of healthy neonatal piglets were found in intraperitoneal (gut, liver, spleen) and extraperitoneal (kidneys, bladder, uterus) organs as well as skeletal muscle and thoracic organs including heart and lung. A prominent site of incorporation was the mucosa of the intestine. Donor WJCs recovered from small intestines 1 week after transplant were viable and exhibited self-renewal after re-culturing in vitro. In view of these results, IP transplanted porcine WJCs can be expected to reach most organs of healthy allogeneic animals without the need for immunosuppression or the need to create inflammatory sites to attract them. Considering the potential issues of pulmonary thrombosis and instant blood-mediated inflammatory reactions reported for intravenously administered cells (discussed above), the peritoneal cavity is worth considering as a transplant site for WJCs. The mucosal locations we observed for allogeneic WJCs, combined with the immune modulation described for WJCs in a variety of preclinical and clinical models, makes them potentially useful for protecting tissues from insults that disrupt homeostasis, and for developing WJCs as delivery vehicles to distribute therapeutics to both healthy tissues and sites of disease.

## Additional files

Additional file 1: Methods for mesodermal differentiation of porcine WJCs. (DOCX 20 kb)

Additional file 2: Fluorescent-dye labeling of cells. Methods used to stain porcine WJCs with PKH26GL and CellVue®NIR815. (DOCX 16 kb)

Additional file 3: **Figure S1.** Adipogenic differentiation of porcine WJCs. Cells stained with LipidTOX Red. (A) Adipogenesis resulted in a morphology change from spindle to enlarged spherical shape with intracellular lipid droplets (red spots) stained with the neutral lipid-specific dye, LipidTOX™ Red. (B) As a negative control, porcine WJCs cultured in growth medium displayed fibroblast-like morphology without expression of stained lipid droplets. Cell nuclei were counterstained with Syto®16 and images captured at 40× magnification with 0.6× digital zoom on a Zeiss 710 confocal microscope. (TIF 1620 kb)

Additional file 4: **Figure S2.** Chondrogenic and osteogenic differentiation of porcine WJCs. (A) Chondrogenesis was induced in porcine WJCs in micromass culture in chondrogenic induction medium. Cell pellets stained positively with Alcian Blue indicating the presence of proteoglycans, a component of the matrix. (B) Osteogenic differentiation produced extracellular calcium deposits that stained bright orange-red with Alizarin red dye. (TIF 7604 kb)

Additional file 5: **Figure S3.** Flow cytometric analysis of surface marker expression on porcine WJCs. Cell suspensions were stained with mouse anti-porcine monoclonal antibodies indicated in filled histograms: CD90 (A), CD44 (B), CD105 (C), CD31 (D), CD45 (E), and SLA-DR (F). The empty histogram is the respective IgG isotype control. The data shown are representative of those obtained in four different experiments. (TIF 1042 kb)

Additional file 6: **Table S1.** Phenotype of porcine WJCs. Percent of porcine WJCs that were positive for CD90, CD44, CD105, CD31, CD45, and SLA-DR. (DOCX 14 kb)

Additional file 7: **Table S2.** SRY-positive samples for each female recipient at 1 week after intraperitoneal transplantation. (DOCX 18 kb)

## Abbreviations

DESS: DMSO/EDTA/saturated NaCl buffer
DIG: Digoxigenin
DMEM: Dulbecco’s minimum essential medium
FBS: Fetal bovine serum
FISH: Fluorescent in-situ hybridization
FM: Freezing medium
IP: Intraperitoneal
IV: Intravenous
MSC: Mesenchymal stromal (stem) cell
PBS: Phosphate-buffered saline
PCR: Polymerase chain reaction
RT: Room temperature
WJC: Wharton’s jelly stem cell
