# Supplementary material for: Porcine Wharton’s jelly cells distribute throughout the body after intraperitoneal injection
Source: Stem Cell Res Ther. 2018 Feb 14;9:38. doi: 10.1186/s13287-018-0775-7 (PMC5813394; doi:10.1186/s13287-018-0775-7)
Supplement: Supplementary file 1 — Methods for mesodermal differentiation of porcine WJCs. (DOCX 20 kb) [file 13287_2018_775_MOESM1_ESM.docx]

### Additional file 1: Methods for mesodermal differentiation of pWJCs.

We used StemPro® kits (Gibco). For adipogenesis, passage 3 WJCs were plated in tissue culture flasks (1x10^4^ cells/cm^2^) and grown in complete growth medium until they reached 80-90% confluence and then the medium was replaced with adipogenesis differentiation medium according to the kit instructions. Culture continued in the differentiation medium for 21 d with medium replaced every 3 d. After 21 d the WJCs were washed 3 times with PBS and fixed with 4% paraformaldehyde solution (30 min at room temperature). The fixed cells were rinsed twice with PBS and LipidTOX™ Red (diluted 1:100) (Invitrogen) added to dye the neutral lipid droplets (fluorescence excitation/emission maxima as 577/609 nm) and incubated (30 min at room temperature). Cell nuclei were counterstained with green-fluorescent nuclear stain, Syto®16 (0.05 µM; Invitrogen, 8 min at room temperature). Cells were mounted on slides with cover slips using glycerol and observed under an inverted Zeiss LSM 700 confocal microscope (LSM 700, Carl Zeiss). After sequential excitation, green and red fluorescent images of the same cell were saved and analyzed by Zeiss Zen 2010 software.

For chondrogenic differentiation, WJCs were cultured in chondrogenesis differentiation according to instructions. WJCs suspended in complete growth medium (1.6 x 10^7^ viable cells/mL) were seeded in 5 µL drops in the center of wells of multi-well plates to generate micromass cultures. After 6 h at 38.5°C, 3 ml of warm chondrogenesis differentiation medium was added to each well and the plate was incubated for 21 d at 38.5°C in a humidified atmosphere of 5% CO_2_. Differentiation medium was removed and the cells were washed 3 times with PBS. Then, cells were fixed with 4% paraformaldehyde (30 min at room temperature), rinsed twice with PBS and stained 30 min (Alcian, 1% w/v in 0.1 N HCl). The stained cells were rinsed 3 times with 0.1N HCl and neutralized with distilled water. Blue staining indicated the synthesis of proteoglycans by chondrocytes.

For osteogenic differentiation early passage (P3) WJCs were seeded (5x10^3^ cells/cm^2^) and cultured in growth medium until 80-90% confluent. The medium was replaced with pre-warmed osteogenesis differentiation medium and WJCs cultured for 21 d with osteogenesis medium replaced every 3 d. Then WJCs were washed 3 times with PBS, fixed (4% paraformaldehyde for 30 min at room temperature), rinsed twice with PBS and 2% (w/v) Alizarin Red S solution (pH 4.2 prepared in distilled water) was added. After incubating 3 min the cells were rinsed 3 times (distilled water) and visualized for Alizarin Red stained mineral deposition.
