# Supplementary material for: Porcine Wharton’s jelly cells distribute throughout the body after intraperitoneal injection
Source: Stem Cell Res Ther. 2018 Feb 14;9:38. doi: 10.1186/s13287-018-0775-7 (PMC5813394; doi:10.1186/s13287-018-0775-7)
Supplement: Supplementary file 2 — Fluorescent-dye labeling of cells. Methods used to stain porcine WJCs with PKH26GL and CellVue®NIR815. (DOCX 16 kb) [file 13287_2018_775_MOESM2_ESM.docx]

**Additional file 2: Fluorescent-dye labeling of cells.**

Expanded adherent cells were harvested from tissue culture flasks using trypsin/EDTA and single-cell suspensions (approximately, 1x10^7^ cells) were washed with serum free medium and precipitated (400 x g). The supernatant was aspirated and the cells were re-suspended and dispersed with 1 ml of Diluent C provided by the manufacturer. Immediately prior to staining, 4x10^-6^ M of either PKH26GL (Sigma-Aldrich) or CellVue®NIR815 (LI-COR Biosciences, Lincoln, NE) dye solution was prepared and 1 ml of cell suspension was added rapidly to 1 ml of dye solution followed by incubating at 25°C for 5 min. The staining reaction was stopped by adding an equal volume of serum and the mixture incubated 1 min. The serum-stopped sample was diluted with an equal volume of complete medium and stained cells centrifuged (400 x g, 10 min at 25°C) and the supernatant removed. After rinsing twice the cells were re-suspended in high-glucose Dulbecco’s Minimum Essential Medium with HEPES at a concentration of 1x10^7^cells/ml.

To verify cell staining and viability, PKH26GL stained cells were stained also with the cell-impermeant cyanine dye, SYTOX® Green dye (0.05 µmol/L; Invitrogen). Fluorescing cells were detected by emition of red and green fluorescence with a microcapillary cytometer (Guava EasyCyte Plus, Millipore). Cells stained with CellVue®NIR815 were verified by plating 2x10^5^ cells in a well of a 96 black-well plate and visualizing with the IVIS Lumina II imaging system (Caliper Life Sciences). Viability of CellVue®NIR815-stained cells was evaluated by dye exclusion using 0.4% trypan blue (Gibco®).
