# Supplementary figures and images for: Porcine Wharton’s jelly cells distribute throughout the body after intraperitoneal injection
Source: Stem Cell Res Ther. 2018 Feb 14;9:38. doi: 10.1186/s13287-018-0775-7 (PMC5813394; doi:10.1186/s13287-018-0775-7)

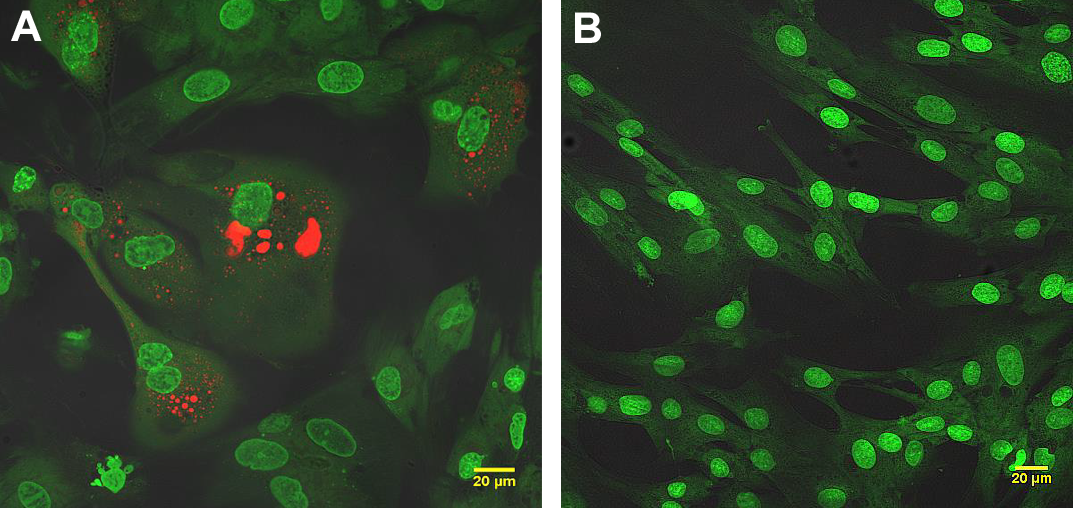

Supplement: Supplementary file 3 — Figure S1. Adipogenic differentiation of porcine WJCs. Cells stained with LipidTOX Red. (A) Adipogenesis resulted in a morphology change from spindle to enlarged spherical shape with intracellular lipid droplets (red spots) stained with the neutral lipid-specific dye, LipidTOX™ Red. (B) As a negative control, porcine WJCs cultured in growth medium displayed fibroblast-like morphology without expression of stained lipid droplets. Cell nuclei were counterstained with Syto®16 and images captured at 40× magnification with 0.6× digital zoom on a Zeiss 710 confocal microscope. (TIF 1620 kb) [file 13287_2018_775_MOESM3_ESM.tif]

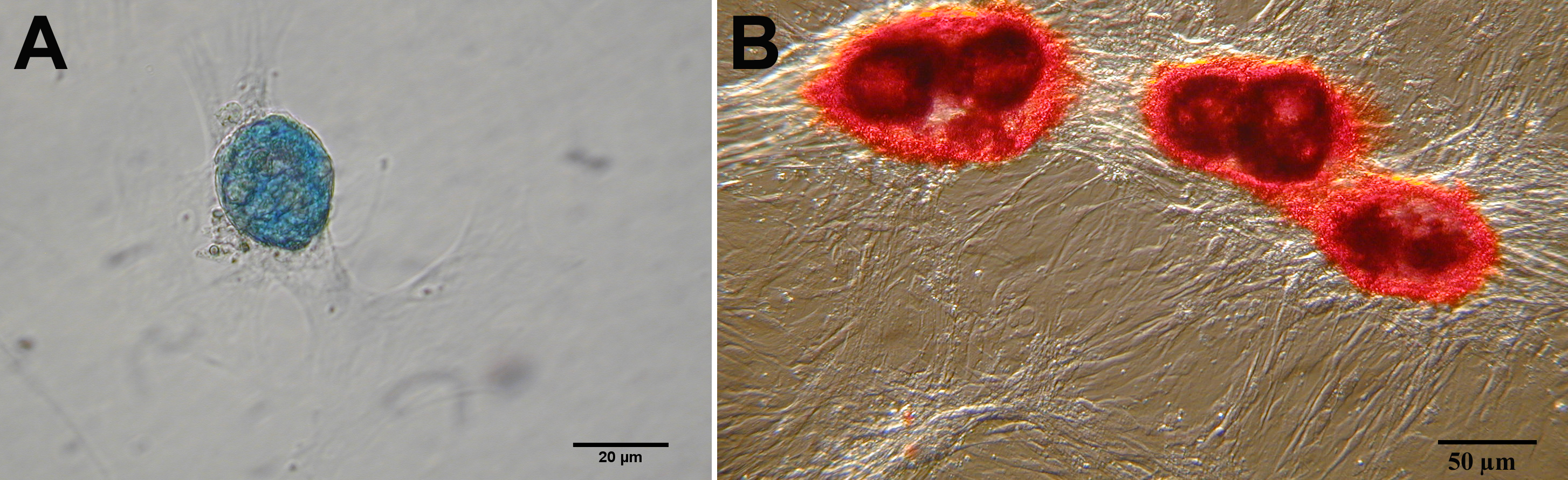

Supplement: Supplementary file 4 — Figure S2. Chondrogenic and osteogenic differentiation of porcine WJCs. (A) Chondrogenesis was induced in porcine WJCs in micromass culture in chondrogenic induction medium. Cell pellets stained positively with Alcian Blue indicating the presence of proteoglycans, a component of the matrix. (B) Osteogenic differentiation produced extracellular calcium deposits that stained bright orange-red with Alizarin red dye. (TIF 7604 kb) [file 13287_2018_775_MOESM4_ESM.tif]

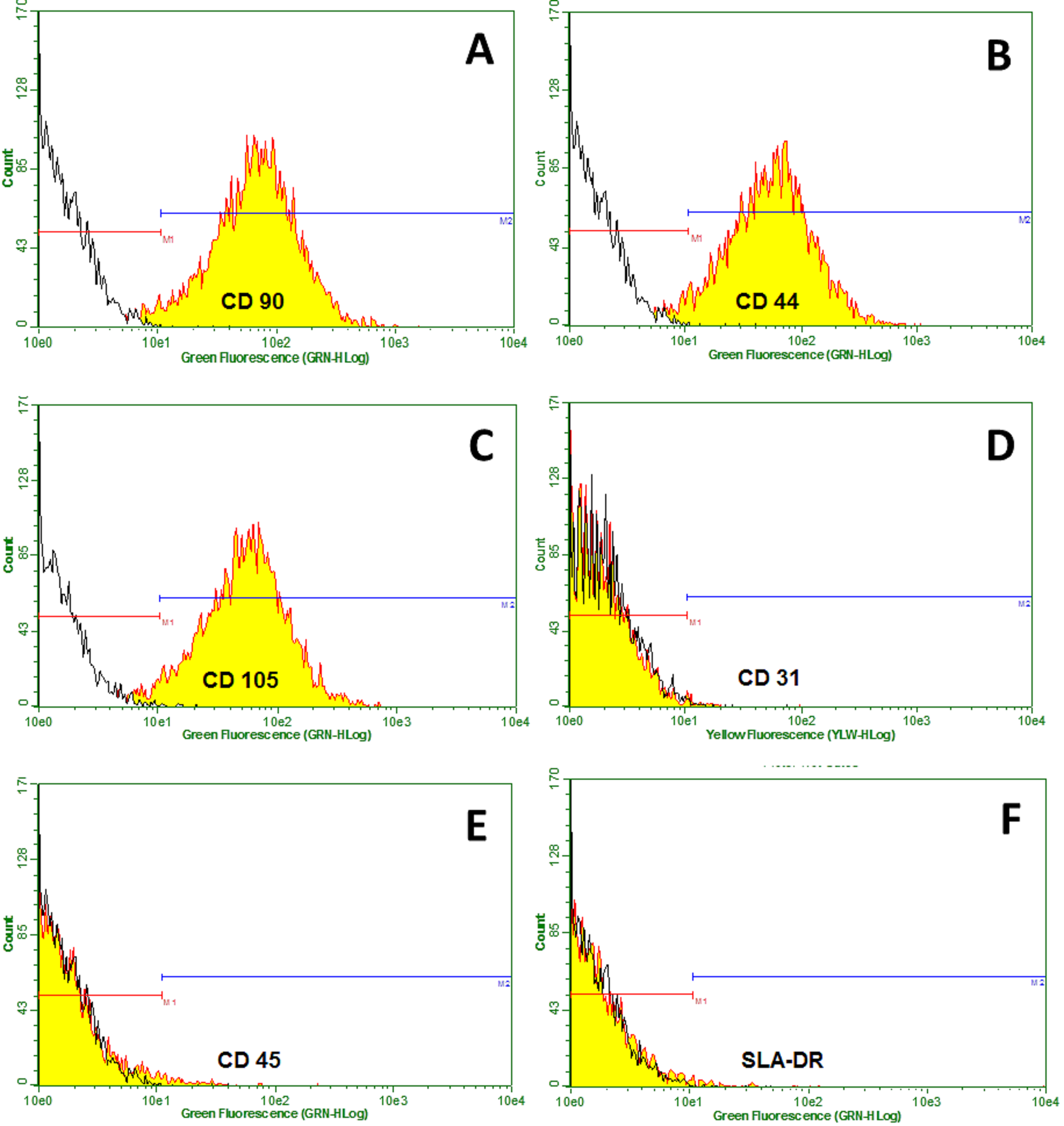

Supplement: Supplementary file 5 — Figure S3. Flow cytometric analysis of surface marker expression on porcine WJCs. Cell suspensions were stained with mouse anti-porcine monoclonal antibodies indicated in filled histograms: CD90 (A), CD44 (B), CD105 (C), CD31 (D), CD45 (E), and SLA-DR (F). The empty histogram is the respective IgG isotype control. The data shown are representative of those obtained in four different experiments. (TIF 1042 kb) [file 13287_2018_775_MOESM5_ESM.tif]
