# Supplementary material for: Porcine Wharton’s jelly cells distribute throughout the body after intraperitoneal injection
Source: Stem Cell Res Ther. 2018 Feb 14;9:38. doi: 10.1186/s13287-018-0775-7 (PMC5813394; doi:10.1186/s13287-018-0775-7)
Supplement: Supplementary file 6 — Table S1. Phenotype of porcine WJCs. Percent of porcine WJCs that were positive for CD90, CD44, CD105, CD31, CD45, and SLA-DR. (DOCX 14 kb) [file 13287_2018_775_MOESM6_ESM.docx]

**Additional file 6: Table S1.**

Table 2.1 Phenotype of pWJCs.

| Antibody | Isolates from Wharon’s jelly | | | | | |
| --- | --- | --- | --- | --- | --- | --- |
|  | 1 | 2 | 3 | 4 | Mean | SD^a^ |
| CD 90 | 94.1^b^ | 93.8 | 92.6 | 90.5 | 92.7 | 1.42 |
| CD 44 | 91.5 | 90.7 | 93.1 | 92.1 | 91.9 | 0.88 |
| CD 105 | 92.8 | 90.5 | 94.2 | 91.3 | 92.2 | 1.42 |
| CD 31 | 0.84 | 0.64 | 0.72 | 0.85 | 0.76 | 0.09 |
| CD 45 | 1.74 | 1.92 | 1.54 | 1.32 | 1.63 | 0.22 |
| SLA-DR | 1.32 | 1.64 | 1.33 | 1.24 | 1.38 | 0.15 |

^a^SD=standard deviation.

^b^% positive cells determined by flow cytometry of 10^6^ WJCs from individual pigs. Cells were at passage 3 when examined.
