# Supplementary material for: Porcine Wharton’s jelly cells distribute throughout the body after intraperitoneal injection
Source: Stem Cell Res Ther. 2018 Feb 14;9:38. doi: 10.1186/s13287-018-0775-7 (PMC5813394; doi:10.1186/s13287-018-0775-7)
Supplement: Supplementary file 7 — Table S2. SRY-positive samples for each female recipient at 1 week after intraperitoneal transplantation. (DOCX 18 kb) [file 13287_2018_775_MOESM7_ESM.docx]

Additional file 7: Table S2.

SRY-positive samples for each female recipient at 1 wk after intraperitoneal (IP) transplantation.

| Age at transplant | 1 d | 1 wk | | 2 wk | 3 wk |
| --- | --- | --- | --- | --- | --- |
| No. of pigs | 2 | 2 | | 2 | 2 |
| Samples examined for each tissue of each pig | 4 | 3 | 3 | | 3 |
| Tissues | No. of positive samples for each pig | | | | |
| Duodenum | 4,4 | 3,2 | | 2,3 | 3,3 |
| Jejunum | 4,2 | 3,2 | | 2,3 | 3,3 |
| Ileum | 3,4 | 3,3 | | 3,3 | 3,3 |
| Cecum | 2,4 | 2,3 | | 3,2 | 3,3 |
| Colon | 3,3 | 2,3 | | 3,3 | 3,3 |
| Esophagus | ND | ND | | ND | ND |
| Stomach | 4,3 | 3,2 | | 3,3 | 3,2 |
| Liver | 4,2 | 2,3 | | 2,3 | 2,2 |
| Spleen | 4,4 | 2,3 | | 3,2 | 2,2 |
| Omentum | 3,4 | 3,3 | | 2,3 | 2,2 |
| Kidney | 2,2 | 2,2 | | 2,2 | 2,1 |
| Pancreas | 3,4 | 2,3 | | 3,2 | 3,2 |
| Mes. lymph nodes | 2.2 | 2,1 | | 1,2 | 2,2 |
| Heart | 3,2 | 2,2 | | 3,1 | 2,2 |
| Lung | 2,3 | 2,2 | | 2,2 | 2,2 |
| Horn of uterus | ND | ND | | 2,2 | 2,2 |
| bladder | ND | ND | | 1,3 | 1,3 |
| Semimembranosus M. | ND | ND | | ND | 2,2 |
| Bone marrow | 2,4 | 2,3 | | 2,2 | 2,2 |

^a^ND = Not done
